# Emergence of Thelaziosis Caused by *Thelazia callipaeda* in Dogs and Cats, United States

**DOI:** 10.3201/eid3003.230700

**Published:** 2024-03

**Authors:** Ranju R.S. Manoj, Holly White, Rebecca Young, Charles E. Brown, Renee Wilcox, Domenico Otranto, Manigandan Lejeune

**Affiliations:** Cornell University, Ithaca, New York, USA (R.R.S. Manoj, H. White, R. Young, M. Lejeune);; Warwick Valley Veterinary Hospital, Warwick, New York, USA (C.E. Brown);; Countryside Animal Hospital, Staatsburg, New York, USA (R. Wilcox);; University of Bari Aldo Moro, Bari, Italy (D. Otranto);; City University of Hong Kong, Hong Kong, China (D. Otranto)

**Keywords:** thelaziosis, parasites, Thelazia callipaeda, eyeworm, zoonoses, One Health, dogs, cats, United States

## Abstract

We report 2 autochthonous feline thelaziosis cases caused by the eyeworm Thelazia callipaeda and discuss the spread among dogs in the northeastern United States. Phylogenetic analysis suggests the parasite was introduced from Europe. Adopting a One Health approach is needed to limit further spread of T. callipaeda *eyeworms in North America*.

*Thelazia callipaeda* eyeworm was considered an exotic parasite in North America until an autochthonous case was reported in a dog from New York, USA, in 2020 (*1*). *T. callipaeda* eyeworm has been reported in countries in East Asia and the Soviet Union, later expanding its geographic range into Europe (*2*,*3*). This zoonotic parasite primarily infects the orbital cavity of its host causing thelaziosis (*3*). The zoophilic secretophagous male fly, *Phortica variegata*, is a *T. callipaeda* vector; flies ingest first-stage *T. callipaeda* larvae from the lacrimal secretions of an infected host and redeposit them as infective third-stage larvae, which eventually complete their life cycle by developing into adult worms (*4*). *P. variegata* flies have been found in Orange and Monroe Counties in New York (*5*,*6*), which has likely promoted the emergence of *T. callipaeda* eyeworm in North America (*4*). Since the *T. callipaeda* infection in a dog reported in New York in 2020, a total of 11 canine cases (6 in New York, 3 in New Jersey, 1 each in Connecticut and Nevada) and 2 feline cases (both from New York) (Figure 1) have been confirmed morphologically at the Cornell Animal Health Diagnostic Center (AHDC) in Ithaca, New York, USA. We describe 2 feline thelaziosis cases and discuss new canine cases in northeastern United States (New York/New Jersey border) during February 2021–December 2022 and One Health approaches to limit spread of this emerging disease in the United States.

**Figure 1:**
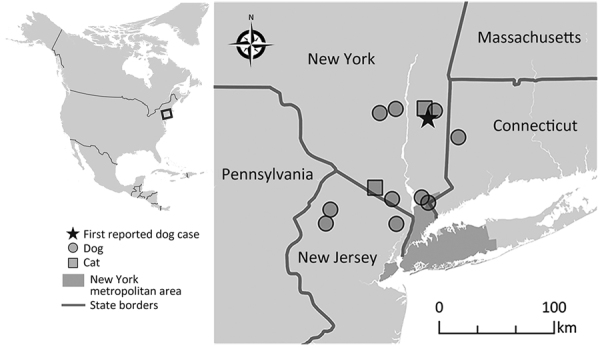
Locations of thelaziosis cases caused by *Thelazia callipaeda* eyeworm in dogs (circles) and cats (squares), New York, New Jersey, and Connecticut, USA. Star indicates the dog case reported in New York in 2020. Inset map indicates the area where *T. callipaeda* infections were reported (box). The dog case reported from Nevada was not included in the map because the travel history for that animal was unknown.

## The Study

Case 1 was in a 16-year-old neutered male, domestic shorthair cat from Greenwood Lake, Orange County, New York, that had been regularly cared for at the Warwick Valley Veterinary Hospital in New York, since October 2019. The animal had a recurrent history of flea infestation, which was managed with selamectin. The cat received routine rabies vaccinations at the clinic and was regularly dewormed with a combination of emodepside (3 mg/kg) and praziquantel (12 mg/kg) applied topically to the skin by the owner. Since June 2021, the animal has been treated for progressive chronic kidney disease. During a visit in April 2022, the cat had crusty lesions on its swollen right eye. Initial treatment with an ophthalmic ointment containing tobramycin resolved the eye infection. In August 2022, the cat manifested squinting, epiphora, and mucus accumulation in the right eye, which did not improve after tobramycin treatment. Detailed examination of the right eye revealed a constricted pupil and an elevated nictitating membrane with 4 thread-like worms, which were recovered mechanically at the clinic by flushing with saline solution. Of the 4 worms collected, 1 intact worm was received at AHDC for identification. The cat did not travel outside of New York. The animal was prescribed an ophthalmic ointment containing neomycin and polymyxin B and a dewormer (combination of emodepside [3 mg/kg] and praziquantel [12 mg/kg]) applied topically to the skin. No relapse was observed after treatment.

**Figure 2:**
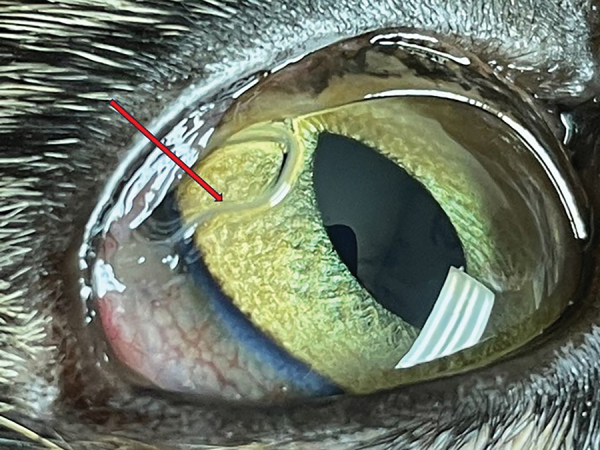
Adult parasites (red arrow) in the bulbar conjunctiva of the left eye of a 2.5-year-old spayed female domestic shorthair cat (case 2) examined in October 2022.

Case 2 was in a 2.5-year-old spayed female, domestic shorthair cat from a multicat household in Clinton Corners, Dutchess County, New York (adopted in Columbia County, New York). The cat did not travel outside of New York and was examined in October 2022 at a pet hospital during a routine rabies vaccination appointment. Ophthalmic examination revealed multiple white thread-like worms on the bulbar conjunctiva of both eyes (Figure 2). The cat had no clinical signs and was prescribed a dewormer (combination of emodepside [3 mg/kg] and praziquantel [12 mg/kg]) applied topically to the skin. Follow-up after 2 weeks revealed the presence of 8 worms, which were manually removed under local anesthesia. Two intact worms were sent to AHDC for identification. The cat was prescribed a combination of imidacloprid (10 mg/kg) and moxidectin (1 mg/kg) applied topically to the skin. Complete recovery was noted during a follow-up visit in November 2022.

At AHDC, we identified 1 male worm from case 1 and 2 female worms from case 2 morphologically as *T. callipaeda* eyeworm, primarily on the basis of transverse cuticular striations. The female worms were 11 and 14 mm long, and the male worm was 8.1 mm long; all 3 had a wide, moderately deep buccal cavity. The number of transverse cuticular striations at the cephalic, midbody, and caudal regions ranged 150–400/mm/region in both male and female worms. In the male worm, the long spicule was ≈2 mm long and the short spicule was 0.1 mm long. The vulval opening in the female worms was anterior to the esophageal/intestinal junction (Appendix Figure 1). 

We performed PCR on 1 female worm sample from feline case 2 and 1 sample from a dog case targeting 12S rRNA, 18S rRNA, and cytochrome oxidase c subunit 1 (*cox1*) using previously described protocols (*7*–*9*). The amplified PCR products for both worm samples were 421 bp for 12S rRNA, 891 bp for 18S rRNA, and 612 bp for *cox1*. We Sanger sequenced the PCR products, edited and aligned the sequences by using BioEdit (https://bioedit.software.informer.com), and compared them with available GenBank sequences by using BLAST analysis (https://blast.ncbi.nlm.nih.gov). We observed 100% sequence identity with corresponding genes available for *T. callipaeda* in GenBank. We deposited the sequences from this study in GenBank under accession nos. OR545549, OR545261, and OR982681. Phylogenetic analysis of the *cox1* sequences revealed clustering as a monophyletic clade with *T. callipaeda* haplotype 1 from Europe (*10*,*11*) (Appendix Figure 2). This study and the previous report on a dog (*1*) reconfirm the possibility that this parasite was introduced from Europe and subsequently spread in the United States.

## Conclusions

The presence of *T. callipaeda* eyeworm in 2 cats and 11 dogs with no travel history outside of the United States suggests that this parasite is emerging in North America. Indeed, a previous study documented the presence of *P. variegata* flies in 2 counties in New York and indicated this fly species is a competent vector for *T. callipaeda* eyeworm, further suggesting an emerging threat by this eyeworm in the northeastern region of the United States (*6*). In addition, a wide variety of wildlife in New York, including coyotes, red foxes, gray foxes, black bears, raccoons, minks, least weasels, striped skunks, cottontail rabbits, and snowshoe hares, might act as potential hosts for *T. callipaeda* eyeworm (*6*); no human cases have been reported from this geographic area. A canine thelaziosis case was also found in the western United States (Nevada), although the travel history is unknown for that dog. Adopting proper diagnosis and surveillance measures is critical to limit the spread of this zoonotic parasite. Studies on control and treatment approaches for dogs suggest mechanical removal of adult and larval *T. callipaeda* nematodes coupled with the administration of diverse deworming drugs is effective (*12*). Because vector control using fly repellents is ineffective (*3*), control of *T. callipaeda* infections mainly rely on diagnosis and timely anthelmintic treatment. The presence of the natural vector, *P. variegata* flies (*4*,*6*), and the potential involvement of the sylvatic cycle promote the spread of this exotic parasite. Most cases in this study were diagnosed in late summer and autumn, which correlates with peak fly activity. Therefore, prophylactic anthelmintic administration coinciding with fly seasons would be an effective control strategy. Furthermore, as indicated in previous reports (*1*,*4*), adoption of a holistic One Health approach will be effective in further limiting the spread of *T. callipaeda* eyeworm in North America.

Appendix Additional information for emergence of thelaziosis caused by *Thelazia callipaeda* in dogs and cats, United States.
