# Supplementary material for: Emergence of Thelaziosis Caused by Thelazia callipaeda in Dogs and Cats, United States
Source: Emerg Infect Dis. 2024 Mar;30(3):591–4. doi: 10.3201/eid3003.230700 (PMC10902549; doi:10.3201/eid3003.230700)
Supplement: Appendix — Additional information for emergence of thelaziosis caused by Thelazia callipaeda in dogs and cats, United States. [file 23-0700-Techapp-s1.pdf]

Article DOI: https://doi.org/10.3201/eid3003.230700

EID cannot ensure accessibility for Appendix materials supplied by authors. Readers who have difficulty accessing Appendix content should contact the authors for assistance.

Emergence of Thelaziosis Caused by *Thelazia callipaeda* in Dogs and Cats, United States

Appendix

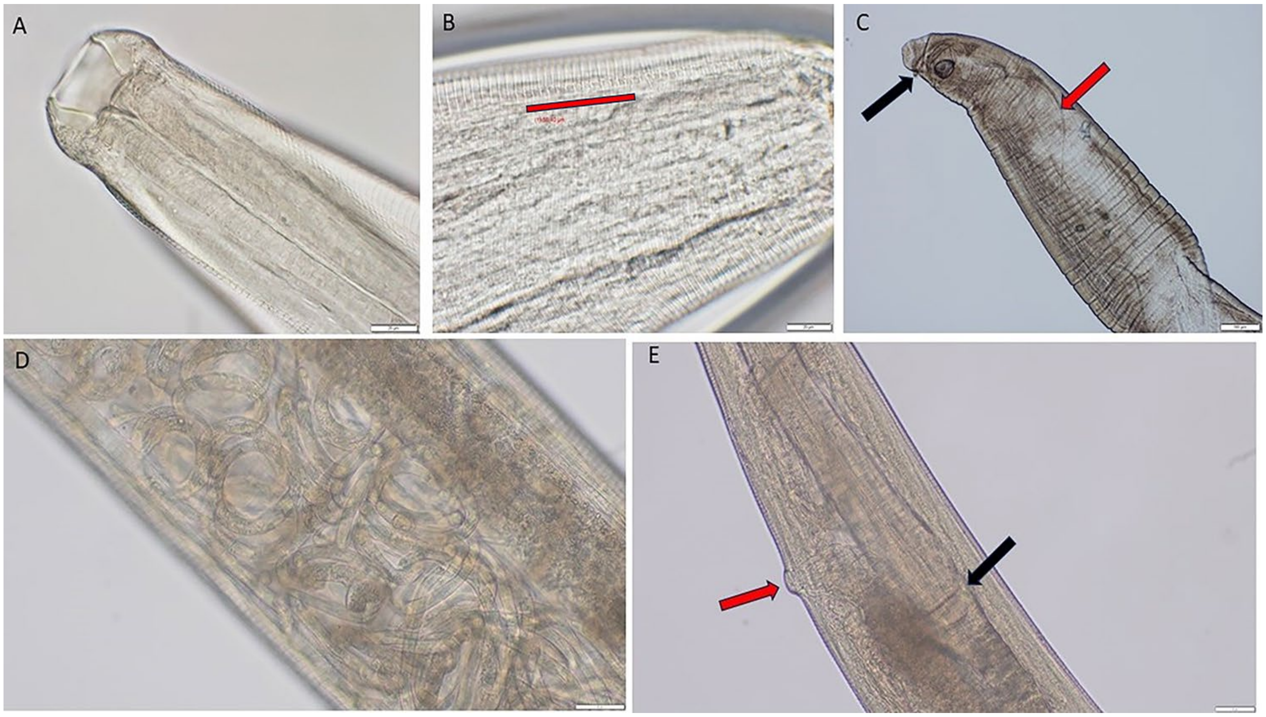

Appendix Figure 1. Morphologic identification of *Thelazia callipaeda*. Specimens were examined under an Olympus compound microscope (BX53) (Olympus, https://www.olympus-lifescience.com), and images were captured with an Olympus DP73 camera. Morphometry was performed with Olympus cellSens software. A) Cephalic end of female worm with a wide, moderately deep buccal cavity. Original magnification ×200. B) Prominent tightly spaced cuticular striations. Red line indicates 50 µm. Original magnification ×200. C) Caudal end of male worm. Red arrow indicates long spicule; black arrow indicates short spicule. Original magnification ×100. D) Uterus of adult worm showing L1 (first) stage of parasite larvae. Original magnification ×500. E) Vulval opening at the esophageal region. Black arrow indicates the esophageal/intestinal junction; red arrow indicates the vulval opening. Original magnification ×100.

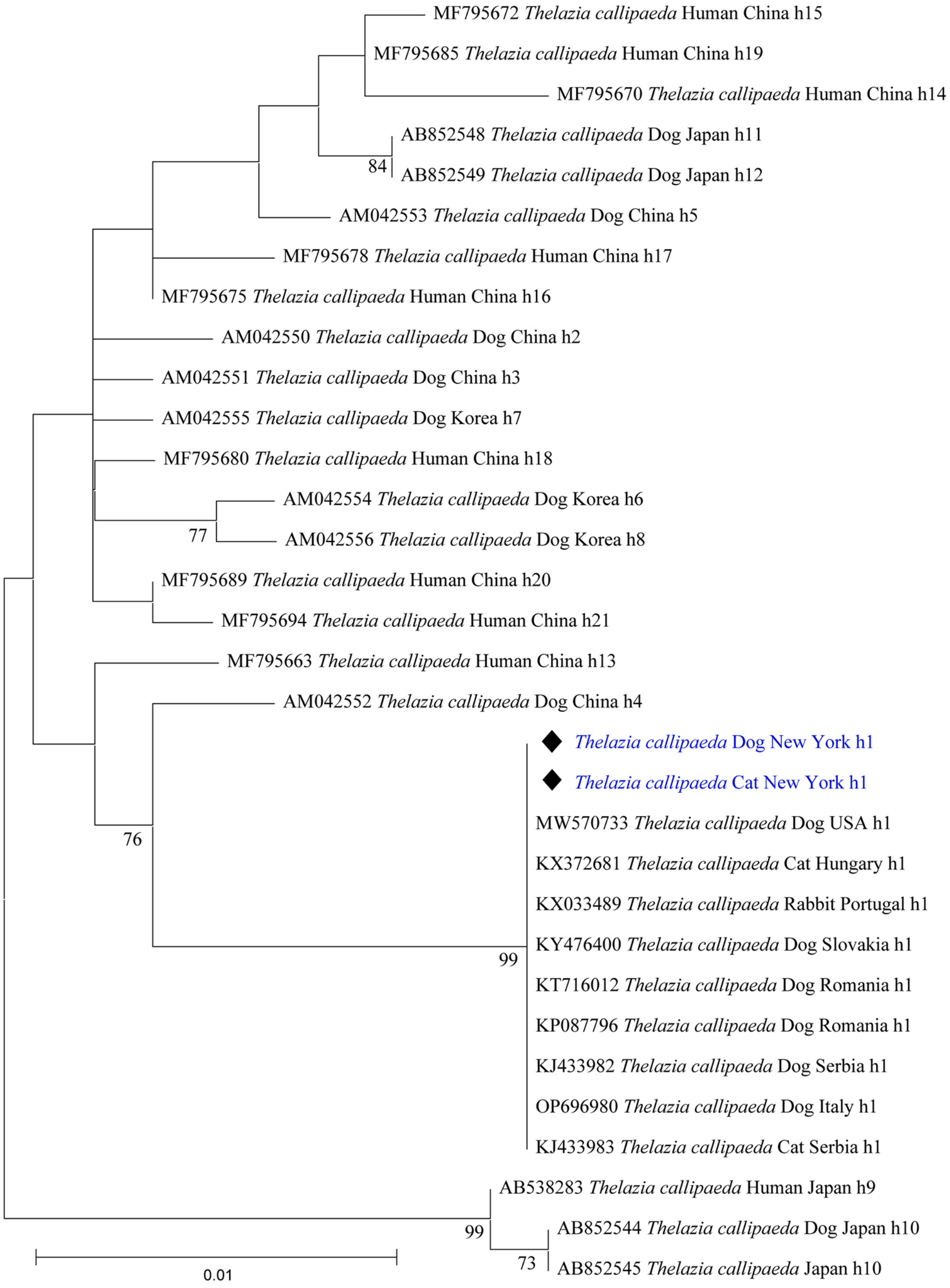

Appendix Figure 2. Phylogenetic analysis of the partial *cox1* gene sequence of *Thelazia callipaeda* detected in this study compared with other *T. callipaeda* haplotypes from GenBank. Analysis was conducted in MEGA11 (*10*, main text) by using the maximum-likelihood method (1,000 bootstrap replications) and the Hasegawa-Kishino-Yano model (*11, main text*). A discrete gamma distribution was used to model evolutionary rate differences among sites. Sequences from this study are highlighted in blue. Scale bar indicates nucleotide substitutions per site.
